# Data on the density of xanthophores in a whole scale of goldfish acclimated to white or black background color

**DOI:** 10.1016/j.dib.2017.08.039

**Published:** 2017-09-01

**Authors:** Kanta Mizusawa, Yutaka Yamamura, Satoshi Kasagi, José Miguel Cerdá-Reverter, Akiyoshi Takahashi

**Affiliations:** a School of Marine Biosciences, Kitasato University, 1-15-1 Kitasato, Minami-ku, Sagamihara, Kanagawa 252-0373, Japan; b Department of Fish Physiology and Biotechnology, Instituto de Acuicultura de Torre de la Sal, Consejo Superior de Investigaciones Científicas (IATS-CSIC), Ribera de Cabanes, Castellón, Spain

**Keywords:** Background color adaptation, Goldfish, Image-processing, Morphological color change, Scale, Xanthophore

## Abstract

The data presented in this article are related to the research article entitled “Expression of genes for melanotropic peptides and their receptors for morphological color change in goldfish *Carassius auratus*” (Mizusawa et al., In press) [1]. This article describes data on the density of xanthophores in the scales of goldfish acclimated to white or black background color. To determine the effects of acclimation history during long-term background color adaptation, fish were transferred from a white tank to a white or black tank and *vice versa* halfway through the acclimation process. To observe xanthophores, the iridophore layer was scraped from the scale and the pteridine/carotenoid pigments were aggregated. The number of xanthophores was calculated after image processing.

**Specifications Table**

**Table:** 

Subject area	Biology
More specific subject area	Fish Physiology
Type of data	Images, table
How data was acquired	Micrographs of scales were acquired using a light microscope (H550L, Nikon, Tokyo, Japan) equipped with a digital still camera (DP25, Olympus, Tokyo, Japan); Image processing and xanthophore counting were performed by using Microsoft ICE 1.4.4 (Microsoft, Redmond, WA), ImageJ 1.44p [2], and GIMP 2.6.11 (http://gimp.org)
Data format	Raw data statistically analyzed
Experimental factors	Scales were obtained from goldfish acclimated to white or black background color
Experimental features	The relationship between the density of xanthophores and the acclimation history during long-term background color adaptation was determined.
Data source location	Kitasato University, Sagamihara, Kanagawa, Japan, 35.54°N, 139.39°E
Data accessibility	The data are available with this article

**Value of the data**

• These data are valuable for researchers studying the physiology of chromatophores in teleosts.
• The method using sequential replacement of experimental animals between different colored tanks during the acclimation period could be useful to determine the effects of acclimation history during long-term background color adaptation.
• The method of image processing will be useful for measuring the distribution of xanthophores in other vertebrates and invertebrates.

## 1 Data

**Fig. 1:**
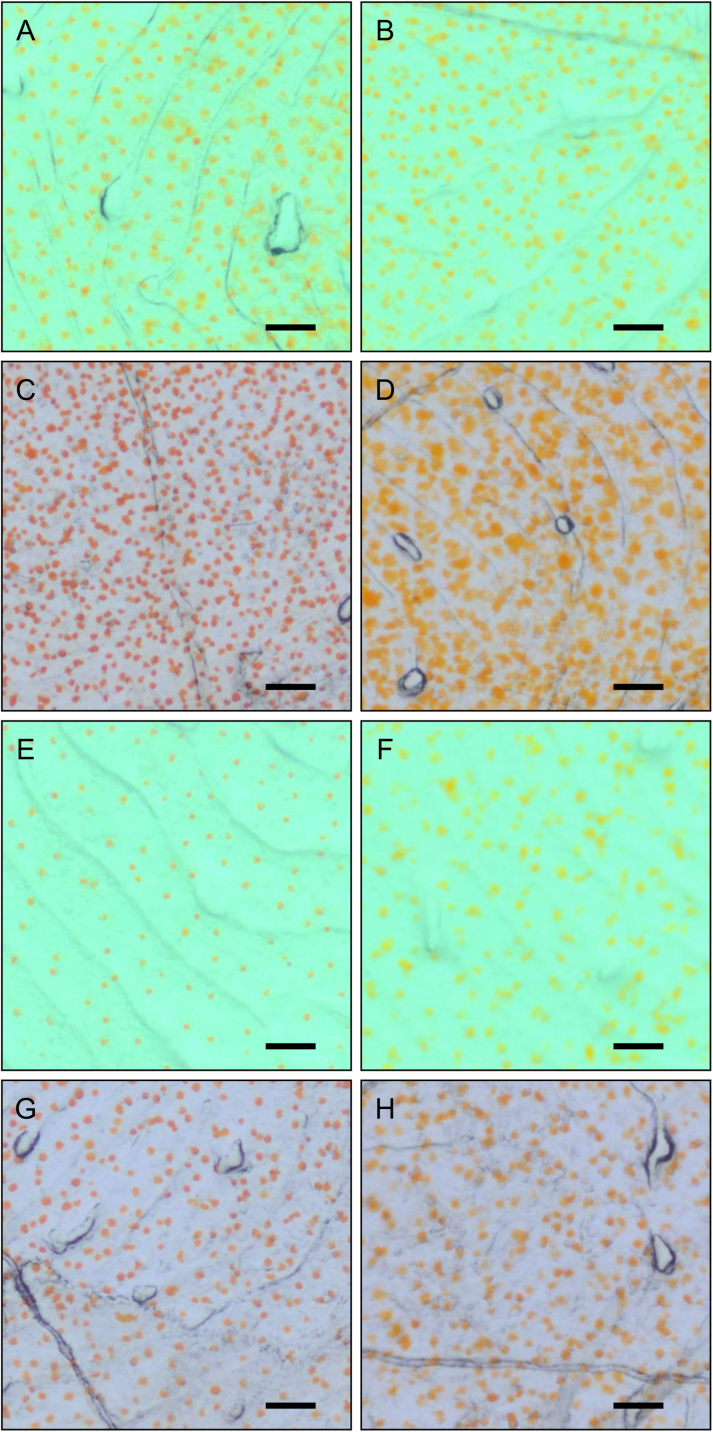
Effects of background color change on the distribution of xanthophores in the scale. The images were taken from the dorsal (A–D) and ventral (E–H) scale of WW fish (A and E), WB fish (B and F), BW fish (C and G), and BB fish (D and H). Scale bar = 0.1 mm.

Fig. 1 shows typical distributions of xanthophores in the dorsal and ventral scales of goldfish reared under scheduled background conditions (21 days under a white or black background prior to 21 days under the opposite background color. Two groups were also transferred to the same background color as controls, see below). Table 1, Table 2 show the number of xanthophores in a whole scale of the dorsal and ventral body, respectively.

**Table 1:** Number of xanthophores in single dorsal scale of goldfish reared under scheduled conditions of background color (cells/scale).

	WW	WB	BW	BB
	3118	2268	8209	2174
	2278	4324	6113	3476
	2975	4607	2562	3694
	1538	3636	6362	2547
	5221	4668	4301	3890
	5277	2942	5459	3574
	3186	3840	2616	2982
	382	3680	7564	1596
	no data	3449	5989	2446
	no data	5587	8191	4780
Average ± S.E.	2997 ± 634^a^	3900 ± 315^a^	5737 ± 315^b^	3900 ± 315^a^

Different letters (“a” and “b”) indicate statistical difference between conditions of background color (*P* < 0.05).

**Table 2:** Number of xanthophores in single ventral scale of goldfish reared under scheduled conditions of background color (cells/scale).

	WW	WB	BW	BB
	1209	2528	4163	3600
	475	2806	2916	2505
	1072	2303	1813	2547
	722	2661	3195	2246
	3145	2448	1886	3271
	570	1744	3959	2368
	714	1717	1789	2668
	1536	2234	1836	1768
	no data	1878	3580	2673
	no data	2946	1837	no data
Average ± S.E.	1180 ± 329^a^	2327 ± 145^b^	2697 ± 325^b^	2627 ± 191^b^

Different letters (“a” and “b”) indicate statistical difference between conditions of background color (*P* < 0.05).

## 2 Experimental design, materials and methods

### 2.1 Acclimation to white or black background color

Goldfish were reared initially in one of four tanks—two white and two black (*n* = 10, body weight = 3.4–5.2 g). On day 21, all fish were anesthetized and the dorsal and ventral scales dissected from the specified areas. Subsequently, fish in white tanks were transferred to either the different white (WW fish) or black tank (WB fish). Similarly, fish in black tanks were transferred to either the different black (BB fish) or white tanks (BW fish). Twenty-one days after the transfer, all fish were anesthetized and the dorsal and ventral scales were collected as before.

### 2.2 Calculation of xanthophore number

**Fig. 2:**
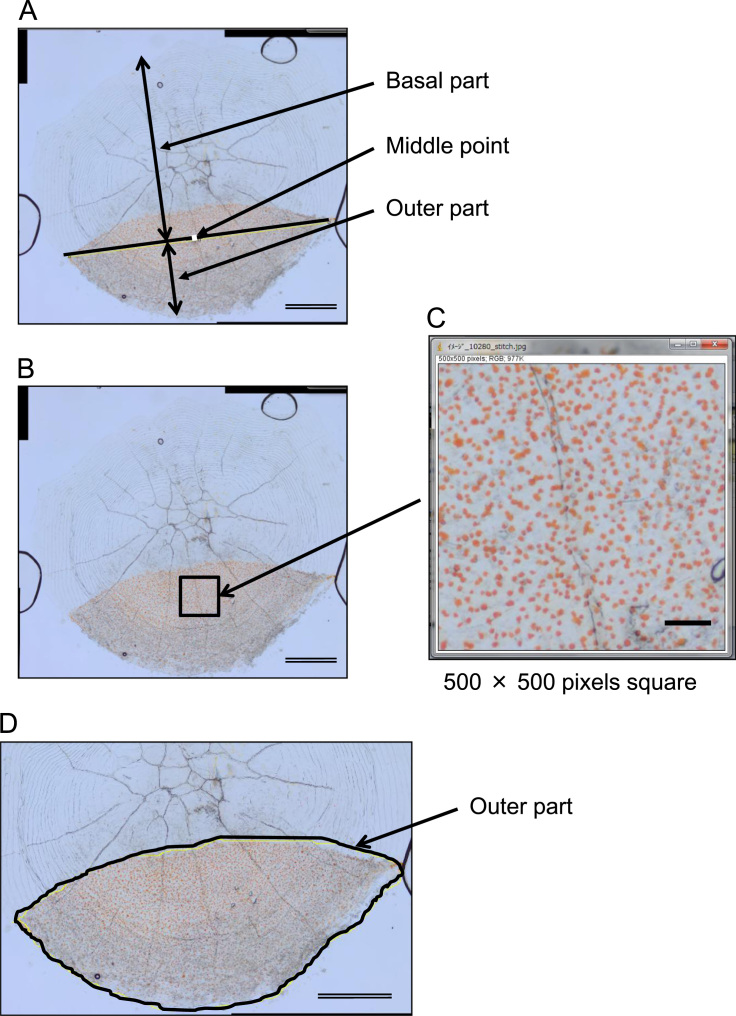
Image analysis for xanthophore counting. Scale bar in single line = 0.1 mm. Scale bar in doubled line = 1 mm.

The scales were observed under a light microscope and the images were processed with multiple software programs to calculate xanthophores in the outer part of the scale [1], as briefly explained below. The iridophore layer on the internal side was scraped with tweezers, and the scales were immersed in 100 mM KCl in Hank's balanced salt solution (HBSS, Thermo Fisher Scientific, Waltham, MA) at 25 °C for 24 h to aggregate the pigments in the xanthophores. The scales were photographed by a light microscope (H550L, Nikon, Tokyo, Japan) equipped with a digital still camera (DP25, Olympus, Tokyo, Japan). Micrographs of all scale parts were assembled to build an image of whole scales. A square image centered on the midpoint of the long diameter of the outer part of the scale (Fig. 2A–C) was processed, and the number of xanthophores in the square was counted. Then, the number of xanthophores in the whole scale was determined based on the density of xanthophores and the square measure based on the outer part of the scale. Microsoft ICE 1.4.4 (Microsoft, Redmond, WA), ImageJ 1.44p [2], and GIMP 2.6.11 (http://gimp.org) were used for image processing.

### 2.3 Statistics

Xanthophore number is expressed as the mean ± standard error values. Differences in values among three or more groups were analyzed by one-way analysis of variance (ANOVA) and the Games–Howell test, a post-hoc multiple comparison test, using StatView 5.0 for Windows (SAS Institute Inc., NC, USA). Normality of all data was confirmed by the Kolmogorov–Smirnov test prior to *t*-test or ANOVA. Significance was determined at the 5% level.
