# Supplementary material for: Data on the density of xanthophores in a whole scale of goldfish acclimated to white or black background color
Source: Data Brief. 2017 Sep 1;14:724–9. doi: 10.1016/j.dib.2017.08.039 (PMC5596328; doi:10.1016/j.dib.2017.08.039)
Supplement: Supplementary file 1 — Supplementary material [file mmc1.docx]

**Conflict of Interest**

KM was funded by JSPS KAKENHI Grant Numbers JP24780192 and JP15K07586 from the Japan Society for the Promotion of Science. JM C-R was funded by AGL2016-74857-C3-3-R from Ministry for Economy and Competitiveness Spain (MINECO).
